# Primary Headaches and Physical Performance: A Professional Youth Female Soccer Team Study

**DOI:** 10.3390/brainsci12121702

**Published:** 2022-12-12

**Authors:** Magdalena Kobus, Elżbieta Żądzińska, Marcos Michaelides, Koulla Parpa

**Affiliations:** 1 Department of Anthropology, Faculty of Biology and Environmental Protection, University of Lodz, 90-237 Lodz, Poland; 2 Biological Anthropology and Comparative Anatomy Research Unit, School of Medicine, The University of Adelaide, Adelaide, SA 5000, Australia; 3 Sport and Exercise Science, UCLan University of Cyprus, Pyla 7080, Cyprus

**Keywords:** headache, migraine, athletes, physical performance, soccer

## Abstract

The vast majority of the research on acute post-traumatic incidents in athletes focuses on migraines. Physical exercise might reduce the frequency of migraines as well as trigger a migraine attack. The objectives of the study were to evaluate primary headache prevalence in professional young female soccer players and to assess whether headaches are related to physical performance. To the best of our knowledge, this is the first study that has considered the relationship between primary headaches and physical performance assessment. The research was conducted in 19 females aged 12–17 from a professional youth soccer academy. Players completed a questionnaire about health status and then performed laboratory physical tests (e.g., cardiopulmonary tests, countermovement and squat jumps, handgrip, sit-and-reach tests). Subsequently, players were separated into a headache group and a headache-free control group. In the analysed group, eight female players (42%) suffered from primary headaches. Among the performance parameters, a significant result was found in terms of flexibility. Females from the headache group had higher results in the sit-and-reach test than females from the control group (*p* = 0.029). Flexibility as well as mobility in migraine patients is an area with significant potential for further investigation, as little research has been conducted to date.

## 1. Introduction

The association between the complex and often nonspecific nature of headaches and sport remains a challenge. The relationship between headaches and sport dates back to classical times. A textbook entitled „De arte gymnastica libri sex” from 1601 is considered to be the first book of sports medicine. The author, Girolamo Mercurialis, was a professor of medicine who became the first sports physician. He drew attention to the health, educational and therapeutic values of physical activity [1]. 

Nowadays, the relationship between headaches and sport has been investigated many times. Physical exercise might have a prophylactic effect on the frequency and severity of migraines and reduce medicine intake [2,3]. However, excessive exercise can worsen pain perception and act as a migraine trigger [4]. Koppen and van Veldhoven reported that 38% of patients experience exercise-triggered migraine attacks during their lifetime [5]. Based on a meta-analysis performed by Lemmens et al., there is moderate evidence that exercise can reduce the number of migraine days by 0.6 per month. The authors pointed out that there are major gaps in the current knowledge, a lack of uniform outcome measures, and that further research is mandatory [6]. So far, the clinical relevance of this finding is low.

There are several pathophysiological explanations of the unclear relationships between physical exercise and migraines. One of the biological mechanisms of exercise that has a therapeutic effect on migraine attacks is increasing anti-inflammatory markers and decreasing pro-inflammatory markers in the brain, which can sensitise the meningeal tissue. On the contrary, during exercise CGRP levels increase and contribute to the release of pro-nociceptive substances. During a migraine attack another possible mechanism for pain aggravation is increased intracranial pressure. The vast majority of people who suffer from migraines avoid routine physical activity during an attack due to an aggravating pain, unlike patients with tension-type headaches. Despite its undeniable association with migraines, CGRP has not been measured during exercise in people who suffer from migraines. Besides the pain pathway, the mechanisms that theoretically trigger attacks through exercise are the impairment of the hypocretin pathway and unfavourable energy metabolism (changes in lactate levels) [7]. 

Exercise-related headaches are strongly associated with sport injuries. Headaches are the most common symptom following concussion in athletes and occur in nearly 93% of sport-related concussions (SRC) cases [8]. Moreover, migraine is the most common phenotype which is associated with trauma-related headache in sport [9]. The role of the pre-traumatic migraine (migraine prior to trauma) in SRC is poorly studied. A few studies have found significant relationships between pre-traumatic migraine and worse SRC consequences [10]. Zemek et al., conducted the largest cohort study of youths with acute concussion, aged 5− < 18 (N = 3063). More than 40% of children with a positive history of migraines experienced a longer duration of persistent concussion symptoms compared to children without a history of migraines (OR 1.9, *p* < 0.001) [11].

Soccer is of interest as it is one of the most popular sports in the world. The prevalence of concussion in young soccer players is higher than in non-contact athletes (50% vs. 9%), but it might be underestimated [12]. Youth athletes may not be aware that they have had a concussion. If so, they may not have reported their symptoms to their parents or coaches and as a result concussion might be overlooked. Later age of first concussion is associated with less severe clinical outcomes (psychological and somatic distress) [13]. Despite the positive impact of sport on wellbeing, adult soccer players are at greater risk of developing depressive symptoms or burnout [14].

The main aim of the study was to assess headache prevalence in young female athletes. The secondary aim was to provide evidence of a relationship between headaches in youth and physical performance. The study involved professional female soccer players, who are at high risk of head injury. 

## 2. Materials and Methods

### 2.1. Participants

The study was conducted in August 2022 and included 19 female players from one of the Cypriot first league team academies. The players were categorised into the headache group and the headache-free control group (according to the International Classification of Headache Disorders, 3rd edition) [15]. The study was approved by the Cyprus National Committee on Bioethics (CNBC, 7 July 2021) and performed according to the guidelines of the Declaration of Helsinki [16]. Written consent was obtained from all the participants and their parents after receiving the detailed study information.

### 2.2. Methods

Information about age and health status (headaches, age of menarche, injuries) were obtained from a questionnaire. The questionnaire data and anthropometric measurements were collected prior to the physical performance testing. The anthropometric measurements (body height and mass) and physical performance measurements were conducted by the professional staff from the Sports Lab, University of Central Lancashire.

### 2.3. Anthropometric and Physical Performance Measurements

Body height was measured with a wall stadiometer (seca GmbH) and body mass was measured with a bioelectrical impedance analyser (BC418MA; Tanita). BMI was calculated as weight in kilograms divided by the square of height in meters. Maximal oxygen consumption (mL/kg/min) was evaluated during an aerobic test on a treadmill (treadmill: h/p/Cosmos Quasar med, H-P-Cosmos Sports & Medical GmbH, Nussdorf-Traunstein, Germany; breath-by-breath analysis: Cosmed Quark CPET, Rome, Italy). Jump height (cm) was measured with 2 types of jumps: a countermovement jump and a squat jump (Optojump). Handgrip strength (kilogram force unit) was measured with a dynamometer (Takei). The sit-and-reach test was performed on a sit-and-reach bench to evaluate flexibility (cm).

### 2.4. Statistical Analysis

All statistical calculations were completed in spreadsheets after the anonymisation of the data. The variables were presented as median and interquartile ranges. The Student t-test was used to assess chronological age, age of menarche, body height, body mass and BMI differences depending on the study group. A multiple regression model was used to analyse the relationship between headache prevalence and physical performance parameters. The statistical analysis of the results was performed in the STATISTICA 13.0 program.

## 3. Results

The headache group included 8 females aged 13–16 (42%), and the headache-free group included 11 females aged 12–17. In the headache group, four females suffered from migraines without aura, three females suffered from coexisting migraines without aura and tension-type headaches and one female suffered from tension-type headaches. The median headache frequency was 5.5 days per month (IQR: 3–6.5). Five females reported attacks related to their periods. All females denied a history of head injury and SRC, which was verified with the medical file inventory.

The median age for female players was 15  years (headache group IQR: 13.5–15, headache-free group IQR: 13–16). Most of the females were post-menarche (headache group: 12.0, IQR: 11.25–12.75, headache-free group: 12.5, IQR: 12.0–13.0), except for one female player (headache-free group). No statistically significant differences in chronological and menarche age were found between the groups (*p* = 0.742, *p* = 0.667). The anthropometric characteristics of the female players per group are presented in Table 1. Among the analysed body size parameters, only body height was significantly different between the groups. The female players from the headache group were shorter than the females from the headache-free group (*p* = 0.040). The multiple regression model showed that among the physical performance parameters, a significant result was found in flexibility (Table 2). The female players from the headache group obtained higher results in the sit-and-reach test (*p* = 0.029). In order to investigate the occurrence of hypermobility, a set of manoeuvres were made (Beighton method). None of the females had a positive result.

## 4. Discussion

The aim of the present study was to assess headache prevalence and provide thorough physical performance tests as evidence of the relationship between headaches and physical performance among young female athletes. In the analysed group, eight female players (42%) suffered from primary headaches, most of them suffered from migraines (seven out of eight). Despite advances in research, the causes of migraine are not fully understood. It seems that migraines have a genetic, prenatal and environmental background and affects prenatal and postnatal phenotypes [17,18,19].

Primary headaches may possibly affect physical performance, but we found a significant result only in the case of one physical ability, flexibility. So far, it has been demonstrated that joint hypermobility has been associated with migraines in females [20]. Moreover, Puledda et al., examined a group of migraine patients, including patients with joint hypermobility syndrome and Ehlers–Danlos syndrome (type 3). The research showed that patients with hypermobility experienced more bothersome symptoms of headaches. In this group, migraine attacks were also more frequent (higher rates of migraine days per month) [21]. Nonetheless, studies on the relationship between flexibility and mobility and headaches are still needed since they have not been conducted in athletes so far. We have highlighted the athletic performance variables that may be related to the headache phenotype, particularly for migraines. Our results suggest broadening research to larger groups of athletes in a variety of sports. 

Possibly there is a gap in the incidence of sport-related concussions in the general population, particularly in the youth athlete population. This might be because young athletes ignore their symptoms and do not report them to adults. We found it important to educate athletes about recognising “the red flags” after potential brain injuries (direct or indirect blow to the head). There are several standardised tools that are easy and quick to implement for medical professionals (e.g., the Sport Concussion Assessment Tool, SCAT5 or the Concussion Recognition Tool, CRT5). The SCAT5 and CRT5 are used in children aged 12 or younger (Child SCAT5) and athletes aged 13 or older [22]. Youth athletes are at higher risk of SRC and experience longer recovery times than adult athletes (i.a., due to maturation) [23]. 

Haran et al., reported that 42% of concussions in young athletes were not managed appropriately on-field and in return-to-play. Among other reasons for this, assessments by qualified personnel were not performed frequently enough (27% of cases) [24]. Moreover, it should be investigated whether the concussion screening interventions address the delayed onset of symptoms [25]. Even a mild head injury or concussion can lead to long-term consequences [26,27]. Delayed effects of these events are common among retired athletes [28]. McCarthy et al. (2022) analysed data from male student athletes to investigate the effects of headaches on neurocognitive function (Immediate Post-Concussion Assessment and Cognitive Testing, imPACT). The study included 960 males who suffered from headaches (mean age: 15.05, 41.3% played soccer) and 5715 headache-free males (mean age: 14.95, 41.1% played soccer), respectively. Headaches were associated with greater fatigue, sadness, emotional lability (irritability), sleep disturbances (sleeping more or less than usual) and lower visual memory outcomes [29]. 

Hence, the risk of traumatic brain injury is higher in contact sports, causes long-term consequences and the recovery from such incidents is longer if the athlete had a history of migraines before the injury [11,12,27]. However, limiting physical activity has a negative effect on medical conditions, both physical and mental. The COVID-19 pandemic has involved temporary restrictive measures and social distancing. The lockdown in Italy resulted in sudden lifestyle changes that have negatively affected the burden of migraines in adult patients. Di Stefano et al., reported significant changes in physical activity, sleep duration and eating habits. In addition, lower physical activity levels were associated with lower sleep quality [30].

In the paediatric population, headaches are the most common disorder. Almost every child and adolescent experiences occasional headaches (75%), while about 10% suffer from recurring headaches [31]. In the young population, the incidence of headaches is significantly higher in females than in males (38.1% in the group aged 12–17 vs. 15.8% in the group aged 6–11) [32]. Chronic headaches cause significant psychosocial disability in school-age individuals. Headaches are one of the three major illness-related reasons for school absenteeism and the most common symptom following concussion in athletes, which might result in severe neurological impairments [8,31]. 

## 5. Conclusions

Educating children, parents and coaches about headaches and raising concussion awareness can be beneficial given their impact on education outcomes and professional careers [33]. Our study suggests increasing communication between coach–family–physician and patient–player.

## Figures and Tables

**Table 1:** Characteristics and differences between anthropometric parameters.

Parameter	Study Group	N	Median	Q_25_–Q_75_	*t*	*p*
Body height (cm)	Headache group	8	157.3	156.5–159.9	2.222	0.040
	Headache-free group	11	164.0	160.0–165.50		
Body mass (kg)	Headache group	8	48.6	47.3–53.3	2.100	0.051
	Headache-free group	11	54.2	50.8–65.0		
BMI (Body mass index) (kg/m^2^)	Headache group	8	19.4	19.0–22.1	1.039	0.313
	Headache-free group	11	21.0	20.5–21.5		

**Table 2:** Characteristics and differences between physical performance parameters.

Test	Study Group	N	Median	Q_25_–Q_75_	*t*	*p*
Squat jump (cm)	Headache group	8	25.5	20.0–26.6	−1.386	0.193
	Headache-free group	11	21.5	17.7–27.5		
Countermovement jump (cm)	Headache group	8	26.6	21.9–30.3	1.300	0.220
	Headache-free group	11	22.6	20.0–28.4		
Sit-and-reach (cm)	Headache group	8	41.0	36.0–43.5	2.519	0.029
	Headache-free group	11	31.0	24.0–39.0		
Right handgrip strength (kgf)	Headache group	8	23.3	22.5–27.5	0.588	0.569
	Headache-free group	11	24.0	22.5–27.0		
Left handgrip strength (kgf)	Headache group	8	22.8	20.8–26.8	−1.070	0.307
	Headache-free group	11	25.0	22.5–26.5		
VO_2_ max (ml/kg/min)	Headache group	8	47.2	45.3–48.3	−0.651	0.528
	Headache-free group	10	47.2	44.5–50.0		

## Data Availability

The datasets used in the current study are available from the corresponding author on request.
